# Special Issue “Cellular and Molecular Mechanisms of Plant Responses to Light, 2nd Edition”

**DOI:** 10.3390/ijms27156617

**Published:** 2026-07-24

**Authors:** Jiangtao Hu, Byoung Ryong Jeong, Qichang Yang

**Affiliations:** 1 Institute of Urban Agriculture, Chinese Academy of Agricultural Sciences, Chengdu National Agricultural Science and Technology Center, Chengdu 610213, China; hujiangtao@caas.cn; 2 Division of Horticultural Science, College of Agriculture and Life Sciences, Gyeongsang National University, Jinju 52828, Republic of Korea

Light is not only the energy source that fuels photosynthesis, but also a highly informative environmental signal that shapes plant development [1,2], metabolism [3,4,5], stress responses [6], and reproductive timing [7,8]. The six papers presented in this Special Issue together offer a multiscale view of plant light biology, spanning small-RNA-mediated signaling, flavonoid and proanthocyanidin metabolism, hormone reprogramming, antioxidant defense, photosystem protection, and circadian regulation of flowering (Figure 1). Collectively, these studies show that light quality affects plant function at multiple regulatory levels, from signaling and gene regulation to metabolic reprogramming, photoprotection, and agronomic performance.

In maize, Fedorin et al. (Contribution 1) revealed that miR165a was a component of phytochrome-dependent signaling under changing light conditions. The authors showed that light-dependent changes in free miR165a were associated with AGO10 binding rather than altered precursor synthesis, and that active phytochrome B played the principal role in regulating mature miR165a abundance. Their model further connected phytochrome signaling with PIF4, AGO10, and the redistribution of miR165a, thereby extending our understanding of how light information can be translated into post-transcriptional gene regulation. This work is noteworthy because it places a conserved microRNA module within a dynamic phytochrome signaling framework and suggests that light-regulated RNA partitioning may be a broader feature of plant adaptive responses.

This Special Issue addressed the metabolic allocation of carbon into specialized compounds with physiological and industrial values. Using spine grape cells, Lin et al. (Contribution 2) demonstrated that overexpression of *VdLAR1* promoted proanthocyanidin accumulation while suppressing anthocyanin biosynthesis, apparently by enhancing catechin competition within the flavonoid pathway. They further showed that light quality modulated this outcome, with white and blue light enhancing the accumulation of proanthocyanidins, catechins, and flavonoids in the transformed lines. This study is significant for two reasons. Firstly, it provides a mechanistic link between a structural gene and branch-point flux within flavonoid metabolism. Secondly, it shows that light quality can be strategically combined with metabolic engineering to direct the production of desirable phytochemicals, a concept of clear relevance for plant cell factories and controlled environment production systems.

In addition, Li et al. (Contribution 3) combined transcriptomics and metabolomics to dissect the effects of far-red and ultraviolet-A supplementation in basil. Far-red light promoted stem elongation and biomass accumulation, while ultraviolet-A affected chlorophyll and flavonoid-related traits. At the molecular level, far-red altered plant hormone signaling, including metabolites and genes associated with gibberellin, cytokinin, brassinosteroid, jasmonic acid, and salicylic acid pathways, whereas ultraviolet-A influenced flavonoid biosynthesis, including genes and metabolites linked to luteolin, apigenin, kaempferol, and related compounds. This paper exemplifies the power of multi-omics in light biology. It moves beyond descriptive phenotype analysis and reveals how spectral cues reshape coordinated networks of hormones, pigments, and secondary metabolites.

The Special Issue also highlights how spectral composition influences plant fitness through redox regulation and physiological acclimation. In alfalfa, Rahman et al. (Contribution 4) compared white, red, blue and combined red–blue light, and found that red light increased oxidative stress markers, whereas combined red–blue light reduced ROS accumulation which was associated with favorable regulation of antioxidant enzymes and genes in the ascorbate-glutathione pathway. These results are especially valuable for forage science and protected cultivation, where optimizing light spectra may improve biomass production and stress resilience simultaneously. Such findings illustrate that light can alter not only growth and morphology, but also the cellular redox environment.

A similar aspect was addressed by Kozuleva (Contribution 5), who examined photoinhibition of photosystem I (PSI) in *Arabidopsis thaliana* and showed that artificial fluctuating light and repetitive short saturating light pulses should not be treated as equivalent stresses. Instead, the evidence supports at least two distinct mechanisms of PSI photoinhibition, involving damage on the acceptor or donor side of PSI, with different protective mechanisms operating under the two treatments. This work sharpens the mechanistic resolution of PSI photoinhibition research and has broad implications for our understanding on how plants cope with dynamic field-like irradiance conditions, where the kinetics of photoprotection can determine whether light becomes beneficial or harmful.

Another strength of this Special Issue is the finding that light spectra is not merely a matter of altering plant morphology, but can be used to shorten generation time and improve the efficiency of breeding systems in controlled environments. Li et al. (Contribution 6) connected spectral regulation with one of the most important agronomic traits in crop breeding, the flowering time. Their rice study showed that far-red light accelerated flowering and implicated the circadian rhythm pathway as a central component of this response. The study further suggested that supplemental far-red promoted developmental progression through coordinated effects on leaf area, photosynthetic efficiency, hormonal homeostasis, and flowering-related gene expression. This contribution is especially relevant because it translates molecular understanding into a practical framework for speed breeding.

Taken together, these six papers reveal three broader trends in current plant light research. First, wavelength-specific effects are highly context-dependent: red, blue, far-red, and ultraviolet-A signals are not interchangeable, and their outcomes depend on tissue, species, and developmental state. Second, light responses are deeply integrated with other regulatory layers, including small RNAs, PIF-centered transcriptional networks, antioxidant metabolism, hormone signaling, and the circadian clock. Third, mechanistic discoveries are increasingly converging with application-oriented goals, especially in plant factories or indoor cultivation systems using artificial light as the sole source of light, horticultural quality improvement, and speed-breeding systems. These studies therefore reflect both the conceptual maturation of plant photobiology and its growing relevance to sustainable crop production.

The studies in this Special Issue provide strong experimental foundations for the next phase of research. Looking ahead, several questions remain open (Figure 2). (1) How general are the miRNA- and vesicle-associated mechanisms uncovered in maize across other crops? (2) To what extent can dynamic light programs, rather than static spectral treatments, further improve antioxidant balance and metabolite composition? (3) How should far-red supplementation be timed to uncouple desirable effects on flowering or biomass increase from potentially unfavorable shade-avoidance traits? (4) And how can knowledge of donor- and acceptor-side PSI protection be integrated into lighting strategies that maximize photosynthetic performance under fluctuating environments?

In summary, this Special Issue provides strong experimental foundations for the next phase of research and development in plant photobiology. We hope that the papers gathered here will stimulate further work at the interface of photobiology, molecular regulation, and controlled environment agriculture.

## Figures and Tables

**Figure 1:**
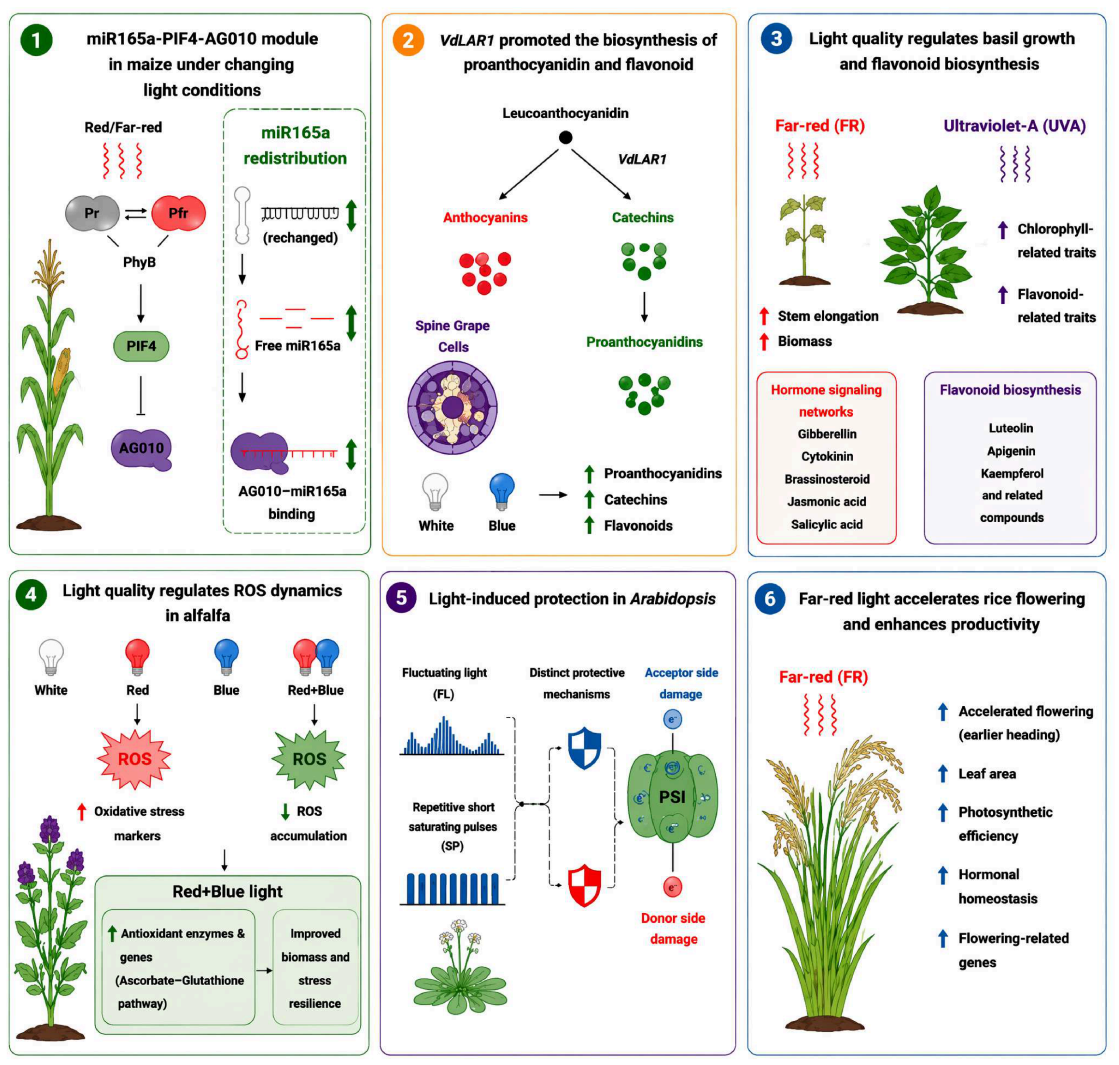
An overview of studies in the Special Issue. Red, green, and purple arrows represent up or down regulation of indicators.

**Figure 2:**
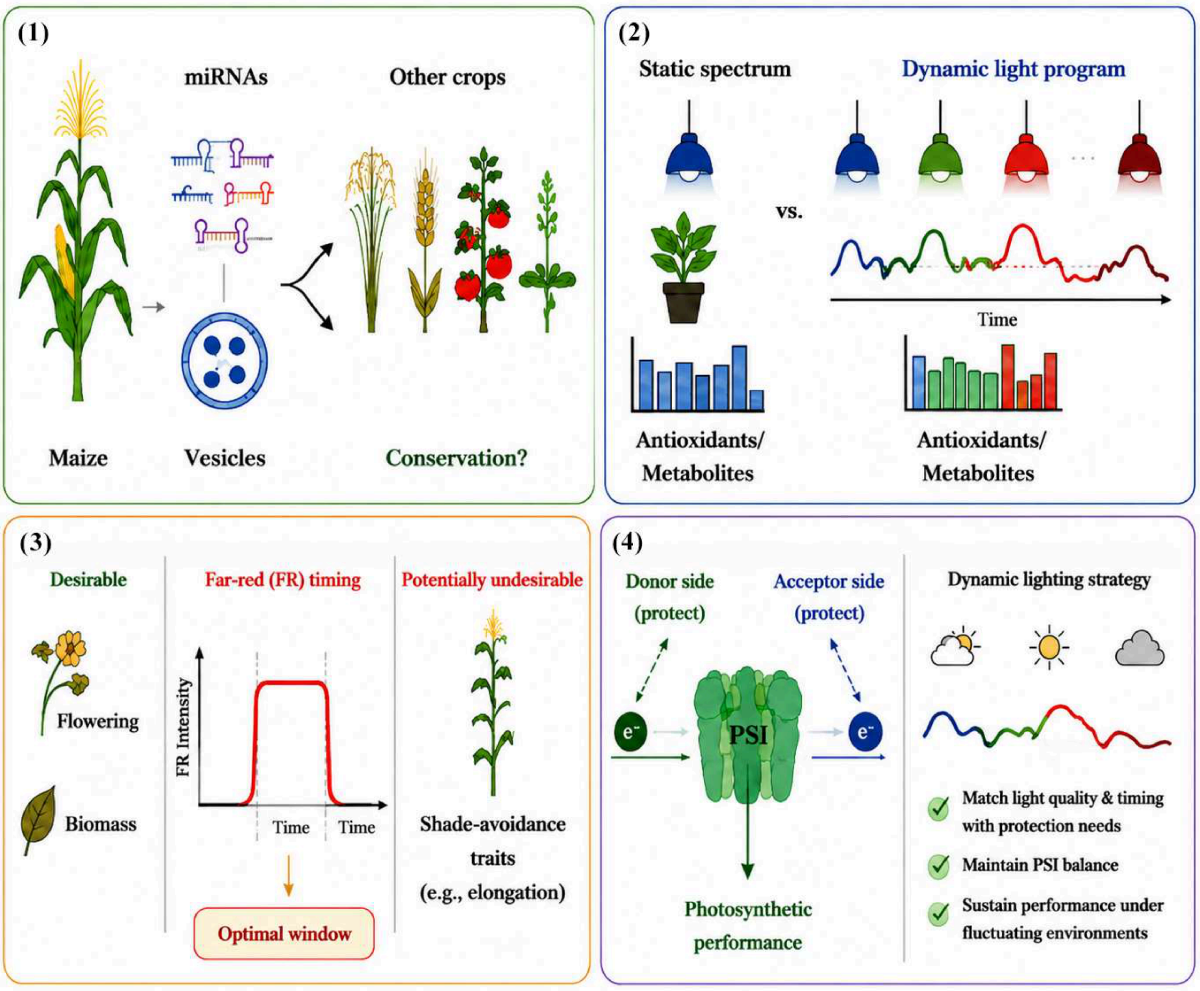
Further questions need to be addressed. (1) Whether the miRNA- and extracellular vesicle–mediated regulatory mechanisms identified in maize are conserved across other crop species. (2) Whether dynamic light regimes can outperform fixed spectral treatments in optimizing antioxidant homeostasis and metabolite profiles. (3) How far-red supplementation can be temporally controlled to promote flowering and biomass accumulation while minimizing undesirable shade-avoidance responses. (4) How donor- and acceptor-side PSI photoprotection mechanisms can be incorporated into lighting strategies to sustain high photosynthetic efficiency under fluctuating light conditions.
